# A continuous fluorescence assay for the characterization of Nudix hydrolases

**DOI:** 10.1016/j.ab.2013.02.023

**Published:** 2013-06-15

**Authors:** Anting Xu, Anna M. Desai, Steven E. Brenner, Jack F. Kirsch

**Affiliations:** a Department of Comparative Biochemistry, University of California, Berkeley, CA 94720, USA; b Department of Plant and Microbial Biology, University of California, Berkeley, CA 94720, USA; c QB3 Institute, University of California, Berkeley, CA 94720, USA

**Keywords:** Nudix, Continuous assay, Fluorescence, Substrate screening, Kinetics

## Abstract

The common substrate structure for the functionally diverse Nudix protein superfamily is nucleotide-diphosphate-X, where X is a large variety of leaving groups. The substrate specificity is known for less than 1% of the 29,400 known members. Most activities result in the release of an inorganic phosphate ion or of a product bearing a terminal phosphate moiety. Reactions have typically been monitored by a modification of the discontinuous Fiske–SubbaRow assay, which is relatively insensitive and slow. We report here the development of a continuous fluorescence assay that enables the rapid and accurate determination of substrate specificities in a 96-well format. We used this novel assay to confirm the reported substrate characterizations of MutT and NudD of *Escherichia coli* and to characterize DR_1025 of *Deinococcus radiodurans* and MM_0920 of *Methanosarcina mazei.* Novel findings enabled by the new assay include the following. First, in addition to the well-characterized hydrolysis of 8-oxo-dGTP at the α–β position, MutT cleaves at the β–γ phosphate bond at a rate of 3% of that recorded for hydrolysis at the α–β position. Second, MutT also catalyzes the hydrolysis of 5-methyl-dCTP. Third, 8-oxo-dGTP was observed to be the best substrate for DR_1025 of the 41 compounds screened.

The Nudix protein superfamily is vast and diverse [1], [2]. It comprises 29,400 members in the Pfam database (version 26.0) [3] (http://pfam.sanger.ac.uk/family/nudix), of which less than 1% have been experimentally characterized. Enzymes from this family have been shown to catalyze a large variety of seemingly distantly related reactions, including messenger RNA (mRNA)^1^ decapping [4], alternative mRNA polyadenylation [5], 3′→5′ RNA exonuclease activity [6], isopentenyl pyrophosphate isomerization [7], and hydrolysis of a large group of nucleoside diphosphate derivatives [2]. Other members are involved in ADP-ribose-responsive transcriptional regulation [8] and in the formation of an ADP-ribose-responding calcium channel [9].

Members of the Nudix family typically contain a 23-amino-acid sequence (Nudix box) of Gx_5_Ex_7_REUxEExGU, where U is usually Ile, Leu, or Val and x represents any amino acid. The EUxEE core residues serve as anchors for an Mg^2+^ ligand that associates with a characteristic pyrophosphate linkage that is common to nearly all Nudix substrates [4].

Although several of the Nudix proteins’ experimentally defined functional assignments are secured by genetic evidence [10] or by high-quality kinetic characterization [11], [12], the vast majority of them have been made by crude enzyme screens. In many cases, the reported values of *k*_cat_/*K*_m_ are too low to inspire confidence that the natural substrate was identified.

Many of the Nudix hydrolases catalyze the hydrolysis of aberrant deoxynucleoside triphosphates efficiently, thereby reducing the extent of incorporation of the corresponding undesired base into DNA. The function of these enzymes is to “sanitize the nucleotide pool”, thereby serving to prevent incorporation of non-canonical bases into DNA [1]. The best-characterized member of this group is MutT from *Escherichia coli*[13]. This gene product exhibits a nearly diffusion controlled *k*_cat_/*K*_m_ value of approximately 10^7^ M^−1^ s^−1^ for the hydrolysis of mutagenic 8-oxo-dGTP; however, it also catalyzes the hydrolysis of the canonical dGTP with a *k*_cat_/*K*_m_ value of approximately 10^3^ M^−1^ s^−1^[14]. This last figure likely represents unavoidable collateral damage due to the structural similarity of the substrates.

The standard assay for those Nudix hydrolases that expose a product phosphate ester or P_i_ is a variant of the classical Fiske–SubbaRow method [15] following treatment of the product, if necessary, with alkaline phosphatase (APase) [12]. This discontinuous assay necessitates removing aliquots during the time course from the reaction mixture. Thus, it is slow, labor intensive, and subject to pipetting error, and it is not optimal for rapid screening of candidate substrates.

We describe here a continuous assay that employs the fluorescently tagged *E. coli* phosphate binding protein (PBP) [16]. It is free of the described limitations of the Fiske–SubbaRow assay and is suitable for substrate and inhibitor screening in a moderate throughput format using 96-well plates.

The enzymes characterized here via this assay are MutT (*E. coli*), NudD (*E. coli*), DR_1025 (*Deinococcus radiodurans*), and MM_0920 (*Methanosarcina mazei*). X-ray or nuclear magnetic resonance (NMR) structures are available for all four enzymes. The first two were well characterized by the Fiske–SubbaRow assay [4] and were selected to validate the new assay. DR_1025 was chosen because *D. radiodurans*, which is highly resistant to radiation damage, has 26 putative Nudix enzymes that likely are responsible for some of the protection [17]. Some substrate screening of the DR_1025 activity has been reported [17]. MM_0920 is a putative Nudix enzyme. A phylogenetic analysis of MM_0920 with 142 biochemically or structurally characterized Nudix proteins has been performed (J.R. Srouji, unpublished work). It was determined that MM_0920 is located in a clade where protein function appears to change frequently. The biochemical characterization of MM_0920 would allow us to infer the function of many other uncharacterized Nudix hydrolases, and it would greatly aid in more accurate function predictions for the entire Nudix family.

## Materials and methods

### Materials

The *E. coli* enzymes MutT (UniProt AC: P08337) [18] and NudD (UniProt AC: P32056) [19] were gifts from Albert Mildvan (Johns Hopkins University School of Medicine), and DR_1025 of *D. radiodurans* (UniProt AC: Q9RVK2) [17] was a gift from Maurice Bessman (Johns Hopkins University). MM_0920 (UniProt AC: Q8PYE2) of *M. mazei* is encoded in the plasmid constructed by the New York SGX Research Center for Structural Genomics and was purchased from the PSI:Biology-Materials Repository (clone ID: MmCD00291907) [20]. The PBP A197C [21]*E. coli* expression strain was a gift from Martin Webb (National Institute for Medical Research, UK). The BL21(DE3) *E. coli* expression strain was purchased from Invitrogen (Carlsbad, CA, USA).

The Malachite Green Phosphate Detection Kit was purchased from R&D Systems (Minneapolis, MN, USA). The Novagen BugBuster protein extraction reagents were obtained from EMD4Biosciences (Gibbstown, NJ, USA). The Gene Jet Plasmid Miniprep Kit was obtained from Fermentas (Glen Burnie, MA, USA). Amicon Ultra-15 and Ultra-4 NMWL 10,000 and 0.22-μm PES syringe membranes were purchased from Millipore (Bedford, MA, USA). Modified nucleotide triphosphates (e.g., 8-oxo-dGTP, 5-methyl-dCTP) were purchased from TriLink Biotechnologies (San Diego, CA, USA). *N*-(2-(1-Maleimidyl)ethyl)-7-(diethylamino)coumarin-3-carboxamide (MDCC), common biochemicals, and enzymes were obtained from Sigma–Aldrich (St. Louis, MO, USA). Fast-performance liquid chromatography (FPLC) for protein purification was performed on a BioLogic DuoFlow 10 workstation (Bio-Rad, Hercules, CA, USA). All liquid chromatography columns were supplied by GE Healthcare (Piscataway, NJ, USA): HiPrep Q FF 16/10 anion exchange, Mono-Q 4.6/100 PE anion exchange, Superdex 75 10/300 GL size exclusion, and PD-10 (Sephadex G-25M) desalting columns. P_i_-sensor assays were performed on either a FluoroMax-4 spectrofluorometer (HORIBA Jobin Yvon, Edison, NJ, USA) or a GENios microplate reader (Tecan, Männedorf, Switzerland). The Fiske–SubbaRow assay was monitored on a NanoDrop 2000c spectrophotometer (Thermo Scientific, Rockford, IL, USA).

### Nudix enzyme purification

MutT was expressed and purified as described by Harris and coworkers [18] with the following changes. The BL21(DE3) cells bearing the pET-MutT plasmid were grown overnight in LB medium at 37 °C and then diluted (1:50) to 2 L and grown at 37 °C for approximately 2 h until *A*_600_ was between 0.5 and 1.0. Isopropyl-β-d-thiogalactoside (IPTG) was added to a final concentration of 0.5 mM to induce protein production for another 2 h. The cells were harvested by centrifugation at 4500*g* and 4 °C for 20 min. The cell pellets were stored at −80 °C. Frozen cell pellets corresponding to 0.5 L of cell culture were lysed with BugBuster reagents as described in the kit protocol. Cell debris was removed by centrifugation at 14,000*g* and 4 °C for 20 min.

MutT precipitates between 30 and 60% (w/v) ammonium sulfate [22]. The pellet was dissolved in 4 ml of 10 mM Tris–HCl (pH 7.6), desalted through two PD-10 columns, and filtered through a 0.22-μm PES syringe membrane. The filtrate was applied to a HiPrep Q FF 16/10 column, equilibrated with 10 mM Tris–HCl (pH 7.6), and eluted with a 0 to 100% gradient of 1 M NaCl. Fractions that contained MutT were collected, combined, concentrated to less than 500 μl using Amicon Ultra-15 Centrifugal Filter Units (Millipore), and loaded on a Superdex 75 10/300 GL column. Eluted fractions that contained MutT were shown to be more than 95% pure by sodium dodecyl sulfate–polyacrylamide gel electrophoresis (SDS–PAGE). These fractions were combined, concentrated to approximately 100 μM with Amicon Ultra-4 Centrifugal Filter Units, divided into 10-μl aliquots, and stored at −80 °C.

NudD was similarly purified. It precipitated between 45 and 80% of ammonium sulfate [23]. DR_1025 precipitated at 2.4 M ammonium sulfate. In addition, 1 mM dithiothreitol (DTT) was included throughout the whole purification procedure of DR_1025 to protect its cysteine residue.

The vector harboring MM_0920 fused with the C-terminal 6-His tag was extracted from the storage strain of the PSI:Biology-Materials Repository (kanamycin resistant, grown in LB medium) using the standard protocol of the Gene Jet Plasmid Miniprep Kit. The plasmid was transformed into BL21(DE3) cells and was induced, harvested, and lysed as described above for MutT. The cell lysate was filtered through a 0.22-μm PES syringe membrane, and the filtrate was applied to a HisTrap HP column equilibrated with 50 mM Tris–HCl buffer (pH 7.6) containing 30 mM imidazole. MM_0920 was eluted with a 0–100% gradient of buffer containing 500 mM NaCl and 500 mM imidazole. MM_0920 fractions were combined and concentrated to less than 500 μl by Amicon filtration. The final preparation of MM_0920 was more than 95% pure as judged from SDS–PAGE. These fractions were combined, concentrated to approximately 100 μM, divided into 200-μl aliquots, and stored at −80 °C.

### Preparation of P_i_ sensor

PBP was expressed and purified as described by Brune and coworkers [21] with the following changes. The supernatant from the sucrose osmotic shock was adjusted to 10 mM Tris–HCl (pH 8.2) and loaded onto a HiPrep Q FF 16/10 anion exchange column equilibrated with the same buffer. The column was washed with 100 ml of 10 mM Tris–HCl (pH 8.2) and eluted with a 100-ml linear gradient from 0 to 250 mM NaCl in 10 mM Tris–HCl (pH 8.2). PBP eluted at approximately 100 mM NaCl. PBP-containing fractions were concentrated to approximately 2 mM by Amicon filtration. The final protein solution was stored in 0.5-ml aliquots at −80 °C. Protein concentration was determined from *A*_280_, assuming a calculated extinction coefficient of 61,880 M^−1^ cm^−1^ as calculated from amino acid composition [24].

The P_i_ sensor was prepared by labeling PBP with a fluorescent tag, MDCC, as described by Webb [25] with the following changes. The 20-mM MDCC solution in *N*,*N*-dimethylformamide (DMF) was prepared immediately prior to the labeling step to avoid precipitation that was observed when the solution had been stored for a long period at −20 °C [25]. The PBP elution was diluted to 100 μM in 10 mM Tris–HCl (pH 8.2) and reacted with 150 μM MDCC (20 mM stock solution in DMF) in a scale of 5 to 50 ml. We found that the extent of labeling was incomplete when the reaction solution was less than 5 ml. The labeling step was performed at room temperature for 45 min in a foil-wrapped tube. The reactant was filtered through a 0.22-μm PES syringe membrane and desalted with a PD-10 desalting column. The filtered desalted sample was loaded on a Mono-Q anion exchange column, which was washed with 10 ml of 10 mM Tris–HCl (pH 8.2), followed by a 20-ml gradient of 0 to 10 mM NaCl. The bright yellow eluate contained the active P_i_ sensor, whereas the inactive P_i_ sensor remained in the column, as reported previously [21]. The protein was concentrated to approximately 1 mM by Amicon filtration and stored in 50-μl aliquots at −80 °C. The characterization of the P_i_ sensor was performed as described by Webb [25]. Typically, 5 μM P_i_ sensor had a linear responsive range to phosphate up to 60% of the sensor concentration.

### Enzyme assays

The P_i_-sensor kinetic assay was performed on either a spectrofluorometer (reaction volume = 500 μl) or a microplate reader (reaction volume = 100 μl). The standard reaction mixture contained 10 mM Tris–HCl (pH 7.6), 1 mM MgCl_2_, 5–10 μM PBP–MDCC (depending on the concentration of background phosphate introduced by the substrate impurities), 0.005 U/ml inorganic pyrophosphatase (PPase) where pyrophosphate was one of the products, or 1–4 U/ml APase where a nucleoside monophosphate was a Nudix enzyme product. Experiments were done in all cases to verify that sufficient coupling enzyme was present to ensure that the rates of reaction were linearly dependent on the concentration of the Nudix hydrolase. The concentrations of the Nudix enzymes and substrates were as follows: 1 nM MutT with 0.2–20 μM 8-oxo-dGTP, 5 nM MutT with 1–20 μM dCTP or 5-methyl-dCTP, 30 nM NudD with 1–6 μM GDP-mannose, and 100 nM DR_1025 with 5–50 μM 8-oxo-dGTP. The mixtures were incubated at 37 °C and monitored continuously for 10 min (GENios: *λ*_ex _= 425 nm, *λ*_em _= 465 nm, gain 50, 100 cycles, 37 °C; FluoroMax-4: *λ*_ex_ = 430 nm, slit width = 1–2.5 nm, *λ*_em _= 465 nm, slit width = 1–5 nm, 37 °C). Data for the linear portions of the initial velocities were fitted by regression (OriginPro 8.1; see below).

The Fiske–SubbaRow assay was performed in 400-μl reaction volumes that contained the same components as listed above minus the P_i_ sensor. The reaction mixtures were incubated at 37 °C for periods of 3–10 min, quenched with 5 mM ethylenediaminetetraacetic acid (EDTA), and stored on ice. The liberated inorganic phosphate was quantitated with the Malachite Green Phosphate Detection Kit (R&D Systems), which essentially employs the colorimetric procedure of Ames and Dubin [15]. Nudix enzyme and substrate concentrations were as follows: 1 nM MutT with 0.2–4 μM 8-oxo-dGTP, 20 nM NudD with 50 μM–2 mM GDP-mannose, and 100 nM DR_1025 with 5–50 μM 8-oxo-dGTP.

The P_i_-sensor screening assay was performed in 96-well plates with reader settings identical to those used in the P_i_-sensor kinetic assays. Although all 96 wells were occasionally monitored on the same plate, 24 or fewer wells were more commonly employed. Substrate concentration was 5 μM and Nudix enzyme concentration was 100 nM, except that 1 nM MutT was used in reactions with dGTP, 8-oxo-dGTP, and 5-methyl-dCTP.

### Data processing

The regression calculations described below were performed with OriginPro 8.1 (OriginLab). P_i_-sensor standard curves were obtained by titrating inorganic phosphate under the same conditions as were used in the experimental reactions. Fluorescence readings were plotted against reaction time, and the linear regions of the plots (relative fluorescence units [RFU]/s) yielded initial velocities, *v*_i_ (μM P_i_/s). Plots of *v*_i_/[E] were fit to the Michaelis–Menten equation or its transformation by nonlinear regression to yield values of *k*_cat_, *K*_m_, and *k*_cat_/*K*_m_. For some reactions, nonlinear regression failed to converge or the standard errors for *k*_cat_ and *K*_m_ were comparable to the fitted values themselves. In these cases, only the *k*_cat_/*K*_m_ ratio was obtained by linear regression with a fixed intercept of zero. Two equivalents of inorganic phosphate are ultimately produced for those reactions that initially yield pyrophosphate in the presence of PPase. The values of *v*_i_ were corrected for this factor. Weighted average values of the kinetic parameters are reported from multiple trials.

## Results

### Description and validation of P_i_-sensor assay

**Fig. 1:**
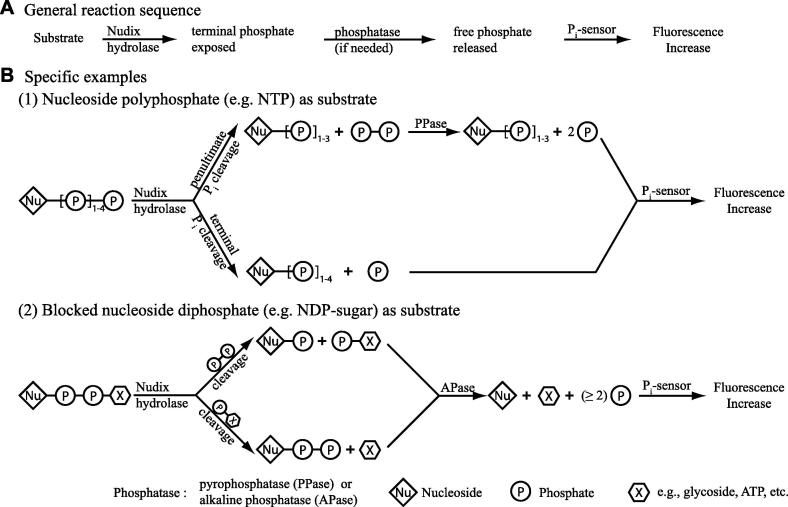
Continuous P_i_-sensor Nudix hydrolase assay. (A) Nudix hydrolase, coupling enzyme (phosphatase), and P_i_-sensor are incubated together with the putative Nudix substrate. Activity is monitored by increase in fluorescence. The choice of coupling enzyme depends on the X moiety (which could be hydrogen, phosphate, or other moieties; see below) of the substrate. The coupling enzyme can be either inorganic pyrophosphatase (PPase) or alkaline phosphatase (APase). (B) Two substrate classes are shown. (1) When X is a hydrogen (i.e., nucleotide diphosphate), a monophosphate (i.e., nucleotide triphosphate), or a polyphosphate (e.g., nucleotide tetraphosphate), Nudix enzyme-catalyzed hydrolysis yields either free phosphate or polyphosphate (e.g., pyrophosphate as shown) as one of the products. In the latter case, pyrophosphatase effects the release of free phosphate. (2) For other X moieties, hydrolysis will not yield free phosphate but will yield at least one phosphate ester as a product. Here, APase catalyzes the hydrolysis to yield free phosphate.

Fig. 1 summarizes the range of Nudix-catalyzed hydrolytic reactions investigated here and diagrams the assay strategy. Nudix enzymes typically catalyze hydrolysis at a diphosphate bond yielding two products, at least one of which has a terminal phosphate group. A coupling enzyme, either PPase or APase, releases at least one equivalent of free phosphate, which is captured by the P_i_ sensor, generating an increase in fluorescence (Fig. 1A). This is recorded continuously to measure initial velocities. No coupling enzyme is required where free phosphate is a direct product of the Nudix hydrolysis (Fig. 1B, part 1, lower branch).

**Fig. 2:**
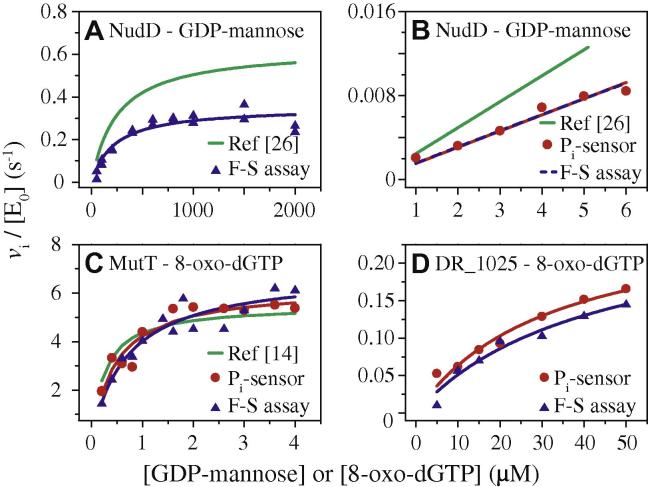
Comparison of *v*_i_/[E_0_] (s^−1^) exhibited by Nudix hydrolases determined by discontinuous assays as measured here and as reported in the literature versus the continuous P_i_-sensor assay. Experimental data are shown as dots or triangles, and fitted data are shown as lines. The values of the kinetic parameters are collected in Table 1. (A) Comparison of Fiske–SubbaRow assay (F–S assay, pH 8.5, 37 °C, blue triangles and solid blue line) and calculated data from (A) in Ref. [26] (pH 8.5, 37 °C, solid green line) for NudD-catalyzed hydrolysis of GDP-mannose. (B) Comparison of P_i_-sensor assay (pH 8.5, 37 °C, red dots and solid red line), calculated data from Ref. [26] (solid green line), and extrapolated data fitting from the Fiske–SubbaRow assay of panel A to low substrate concentration (blue dashed lines) for NudD-catalyzed hydrolysis of GDP-mannose. (C) Comparison of P_i_-sensor assay (pH 7.6, 37 °C, red dots and solid red line), Fiske–SubbaRow assay (pH 7.6, 37 °C, blue triangles and solid blue line), and calculated data from Ref. [14] (pH 8.0, 30 °C, solid green line) for MutT-catalyzed hydrolysis of 8-oxo-dGTP. (D) Comparison of P_i_-sensor assay (pH 7.6, 37 °C, red dots and solid red line) and Fiske–SubbaRow assay (pH 7.6, 37 °C, blue triangles and solid blue line) for DR_1025-catalyzed hydrolysis of 8-oxo-dGTP. Coupling enzymes, where used, are listed in Table 1.

Fig. 2 compares kinetic data recorded by the discontinuous classical Fiske–SubbaRow assay with those yielded by the continuous Pi-sensor assay for three Nudix hydrolases. The results from the two methods are in excellent agreement when the measurements were carried out with the same batch of enzyme. Our preparation of NudD, which catalyzes the hydrolysis of GDP-mannose, was approximately 50% less active than that reported in the literature [26]. The kinetic constants obtained here for the MutT-catalyzed hydrolysis of 8-oxo-dGTP are in agreement with reported values [14], although the experimental conditions differed slightly. These results establish that the continuous P_i_-sensor assay can replace the discontinuous assay, thereby enabling broader screening of potential Nudix substrates and more facile determination of kinetic parameters.

### Substrate screening

**Fig. 3:**
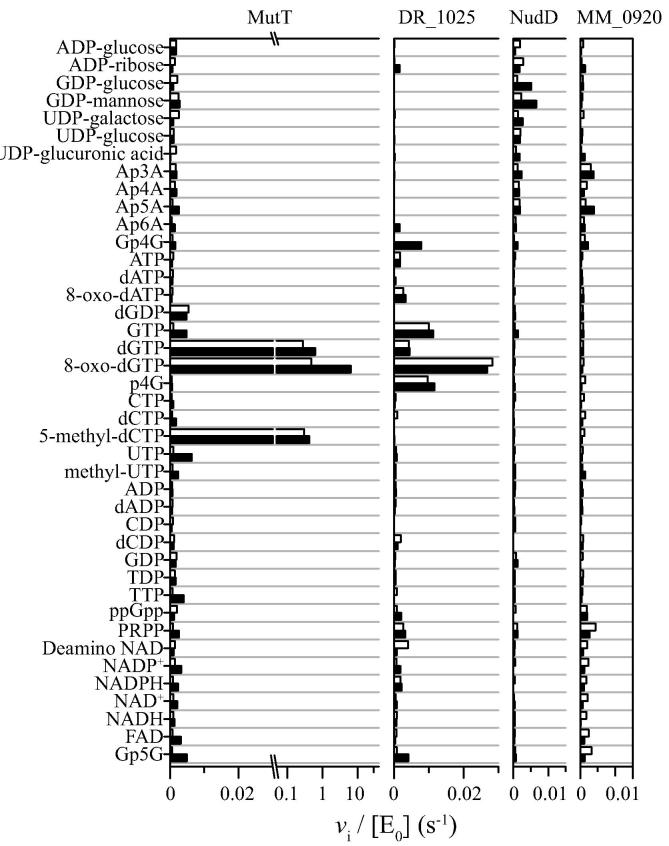
Substrate screening (*v*_i_/[E_0_] in s^−1^) of four Nudix hydrolases (MutT, DR_1025, NudD, and MM_0920) against a selected 41-compound library of reported Nudix substrates by P_i_-sensor assay. Each reaction was carried out with 5 μM substrate at pH 7.6 and 37 °C both in the presence (solid bar) and in the absence (empty bar) of coupling enzyme (see Fig. 1). Reactions of MutT with dGTP, 8-oxo-dGTP, and 5-methyl-dCTP were performed in a single run with 1 nM MutT. All other reactions were assayed with 100-nM Nudix hydrolases. The horizontal axis for MutT after the break is in log scale.

A screening library of 41 commercially available putative substrates was assembled from compounds that had been shown to be active with one or more Nudix hydrolases (J.R. Srouji, J.F. Kirsch, and S.E. Brenner, unpublished work). Fig. 3 shows results of the substrate screening of four Nudix hydrolases: MutT, DR_1025, NudD, and MM_0920. Each substrate was assayed twice: in the presence and absence of PPase or APase as appropriate.

8-Oxo-dGTP is the most reactive MutT substrate (Fig. 3), a result that is consistent with the literature [13]. It is followed by dGTP, which is approximately 10% as reactive as 8-oxo-dGTP, a result that is also in accord with the reported value [27].

Two novel results were uncovered by the screening process: (i) the rate of 5-methyl-dCTP hydrolysis is also approximately 10% of that exhibited by 8-oxo-dGTP, and (ii) the rate of phosphate liberation from 5 μM 8-oxo-dGTP in the absence of added PPase was approximately 10% of that observed in its presence, indicating that MutT catalyzes hydrolysis of 8-oxo-dGTP at both the α–β and β–γ positions.

The order of substrate reactivity of DR_1025 is 8-oxo-dGTP > p_4_G ≈ GTP > Gp4G > dGTP (Fig. 3). This confirms the previously reported DR_1025 activity against dGTP [17]. The reported Ap_4_A hydrolysis [17] was not detected in the screening but was confirmed in a detailed kinetics study (Table 1). The apparent preference for GTP over dGTP noted in the screening experiment was not confirmed in the subsequent analysis (Table 1). The experimentally determined kinetic parameters for the DR_1025-catalyzed hydrolysis of dGTP are essentially the same in the presence and absence of PPase, indicating exclusive cleavage at the β–γ bond (Table 1). NudD shows highest activity toward GDP-mannose and GDP-glucose (Fig. 3), a result that is consistent with the literature [11], [23]. Substrate screening of MM_0920 failed to identify a candidate molecule. A structure similarity search using the DALI server [28] showed that MM_0920 has overall structural similarity with MutT (PDB: 3GRN and 3A6U, with a *Z* score of 16.2), so we tried carefully to measure activity toward 8-oxo-dGTP. The *k*_cat_/*K*_m_ value is very low (Table 1) and is increased approximately 2-fold when the assay pH is raised to 9.0 (data not shown).

### Novel substrate specificities and cleavage pattern of MutT

**Fig. 4:**
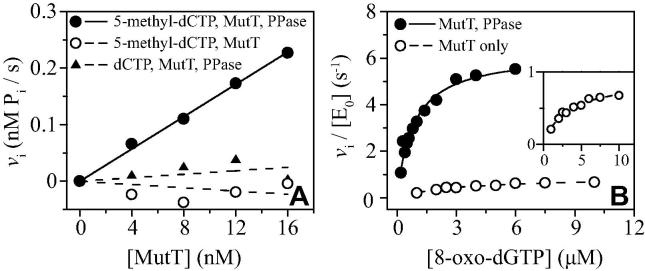
Novel MutT catalytic activities. The reactions were monitored by P_i_ sensor at pH 7.6 and 37 °C. (A) Hydrolysis of 5 μM 5-methyl-dCTP (circles) and dCTP (triangles). Initial velocities, *v*_i_ (nM P_i_/s), are plotted against MutT concentration (nM) in the presence (solid circles and triangles) or absence (empty circles) of PPase. The background (no MutT) activities of 0.23 nM P_i_/s (solid circles and triangles) and 0.36 nM P_i_/s (empty circles) were subtracted from each data point. (B) Hydrolysis of 8-oxo-dGTP either in the presence (solid circles and solid line) or in the absence (empty circles and dashed line) of PPase.

The substrate screening results revealed that MutT has significant activity toward 5-methyl-dCTP. An expanded investigation of this reaction showed that the *v*_i_/[E_0_] values in the presence of PPase are substantially greater than those recorded in its absence, indicating that MutT cleaves mainly the α–β pyrophosphate bond of 5-methyl-dCTP (Fig. 4A). The *k*_cat_/*K*_m_ value for this reaction is approximately 0.01% for that of 8-oxo-dGTP hydrolysis (Table 1), and no rate saturation was observed up to 20 μM substrate (data not shown). MutT reacted only with 5-methyl-dCTP in an activity screening with dCTP, 5-Me-dCTP, 5-OH-dCTP, 5-MeOH-dCTP, 2’-O-Me-dCTP, and N4-Me-dCTP (data not shown). The rate of phosphate release from a 5-μM solution of 5-methyl-dCTP is linear for at least 10% of the reaction course (data not shown), a result that argues that the observed activity is not due to an impurity. The purity of the compound was judged to be approximately 95% by electrospray ionization mass spectrometry (ESI MS; data not shown).

MutT predominantly catalyzes the hydrolysis of 8-oxo-dGTP at the α–β pyrophosphate bond (see Ref. [18] and Table 1); however, the substrate screening experiments showed modest hydrolytic activity for dGTP and 8-oxo-dGTP in the absence of PPase (Table 1). The *k*_cat_/*K*_m_ value for the β–γ hydrolysis is approximately 3% of the α–β cleavage reaction rate of 8-oxo-dGTP (Table 1).

## Discussion

### Comparison of P_i_-sensor and Fiske–SubbaRow assays

The major advantage of the P_i_-sensor assay introduced here for Nudix hydrolases is that it continuously monitors reaction progress and greatly reduces the time and materials required to determine initial velocities (*v*_i_) relative to the Fiske–SubbaRow assay. It can be performed in 96-well plates and, thus, is suitable for simultaneous analysis of several enzymes and substrates and for inhibitor discovery. The accurate determination of *k*_cat_/*K*_m_ values requires high-quality data for *v*_i_ at [S] < *K*_m_. Such data are more readily obtained in a continuous versus discontinuous assay.

The P_i_-sensor assay can distinguish 0.05 μM P_i_ (Fig. 4A) when monitored continuously. In comparison, the lower detection limit of the Fiske–SubbaRow assay is 1 μM [15], [29]. However, the high sensitivity of the P_i_-sensor assay makes it susceptible to traces of inorganic phosphate present in many of the test molecules. A typical assay solution might contain 5 μM P_i_ sensor and 100 μM substrate. Thus, even a small percentage of contamination with phosphate would deplete the P_i_ sensor. This issue might be remedied by adding, for example, unlabeled PBP to the substrate, followed by separation of the PBP–P_i_ complex from the substrate prior to assay. Alternatively, the concentration of the P_i_ sensor can be increased and the fluorescence change may be monitored at a wavelength that is off the emission maximum or by reducing the slit widths. These measures would also alleviate inner filter issues should they be problematic. Fortunately the objective of most screening assays is the determination of *k*_cat_/*K*_m_ values, which is readily accomplished in low substrate concentrations where phosphate contamination is minimal.

### Range of applicability of the assay

Although the P_i_ sensor assay can be employed to monitor the vast majority of known Nudix substrates, it is not universally applicable. For example, the mouse protein RP2p has been shown to be a Nudix hydrolase with acyl-CoA (coenzyme A) diphosphatase activity [30]. APase would, however, likely cleave the unblocked 3′ phosphate of CoA (and its derivatives), releasing free phosphate and, thus, compromising the P_i_-sensor assay. Some cleavage patterns of nucleotide tetra- or pentaphosphate might also escape detection. The protein IalA from *Bartonella bacilliformis* has been shown to hydrolyze adenosine pentaphosphate (p_5_A) to ATP and pyrophosphate [31]. This reaction falls into B.1 of Fig. 1, which uses PPase as the coupling enzyme. However the P_i_-sensor assay would fail to detect cleavage at the β–γ pyrophosphate bond, giving ADP and inorganic triphosphate as products. These limitations, of course, also pertain for the discontinuous assay.

### 5-Methyl-dCTP hydrolase activity of MutT and its possible physiological role

5-Methylcytosine in DNA is a source of mutation [32]. Interestingly, MutT has low but distinguishable hydrolytic activity toward 5-methyl-dCTP in the presence of PPase. The hydrolysis is undetectable when the substrate is dCTP or when PPase is omitted. These observations suggest that MutT cleaves at the α–β phosphate bond of 5-methyl-dCTP. Another *E. coli* enzyme, NudG, when assayed at pH 9.1 and 37 °C, has a reported *k*_cat_/*K*_m_ value of 3 × 10^5^ M^−1^ s^−1^ for 5-methyl-dCTP [33]. This figure is approximately 100-fold higher than that found here for the MutT-catalyzed hydrolysis rate constant. The relative contribution to the in vivo hydrolysis rate of this aberrant NTP is, thus, a function of enzyme abundance. The possibility that our MutT sample is contaminated by approximately 0.5% NudG, which would generate an apparent *k*_cat_/*K*_m_ value similar to that reported in Table 1 for 5-methyl-dCTP, was excluded, because our mass spectroscopy result of MutT indicates that NudG, if any, was presented at less than 0.1% (data not shown).

### β–γ Cleavage in MutT-catalyzed hydrolysis of 8-oxo-dGTP

The predominant cleavage site in the MutT-catalyzed hydrolysis of 8-oxo-dGTP is at the reported α–β position [18]. Such activity requires PPase for detection by the P_i_-sensor assay. A small rate of cleavage over the MutT minus control was recorded in the absence of PPase, indicating that β–γ hydrolysis occurs at approximately 3% of the α–β site cleavage. The significance of this observation is not known. Minor alternate cleavage patterns would likely escape detection in most assays of nucleoside triphosphate hydrolase activity, so it could be a fairly common phenomenon. Alternatively, the evolutionarily acquired promiscuity of the substrate specificity of the Nudix enzyme family may restrict, to some extent, the precision of placement of the catalytic residues near the scissile bond.

## Figures and Tables

**Table 1:** Kinetic parameters for Nudix hydrolase-catalyzed reactions

Nudix hydrolase	Substrate	Assay	pH	Phosphatase	*k*_cat_ (s^−1^)	*K*_m_ (μM)	*k*_cat_/*K*_m_ (M^−1^ s^−1^)
MutT	8-Oxo-dGTP	F–S assay	7.6	PPase	6.9 ± 0.3	0.53 ± 0.09	(9.3 ± 1.1) × 10^6^
			5.5^a^	0.26^a^	2.1 × 10^7^^a^
Pi sensor	7.6	PPase	5.5 ± 0.1	0.28 ± 0.03	(8.9 ± 0.6) × 10^6^
Pi sensor	7.6	None	0.89 ± 0.04	3.0 ± 0.4	(3.0 ± 0.2) × 10^5^
8-Oxo-GTP	P_i_ sensor	7.6	PPase	11.2 ± 0.9	0.9 ± 0.2	(1.3 ± 0.2) × 10^7^
5-Methyl-dCTP	P_i_ sensor	7.6	PPase	ND	ND	(2.8 ± 0.1) × 10^3^
dCTP	P_i_ sensor	7.6	PPase	ND	ND	240 ± 200

NudD	GDP-mannose	P_i_ sensor	8.5	APase	ND	ND	(1.5 ± 0.1) × 10^3^
F–S assay	8.5	APase	0.35 ± 0.02	220 ± 60	(1.6 ± 0.3) × 10^3^
			0.63^b^	250^b^	2.5 × 10^3^^b^

DR_1025	8-Oxo-dGTP	P_i_ sensor	7.6	None	0.18 ± 0.01	26 ± 1	(4.4 ± 0.1) × 10^3^
F–S assay	7.6	None	0.29 ± 0.04	28 ± 7	(6.8 ± 0.8) × 10^3^
8-Oxo-GTP	P_i_ sensor	7.6	None	0.13 ± 0.01	22 ± 5	(5.9 ± 0.8) × 10^3^
dGTP	P_i_ sensor	7.6	PPase	ND	ND	860 ± 20
P_i_ sensor	7.6	None	ND	ND	950 ± 40
GTP	P_i_ sensor	7.6	None	ND	ND	820 ± 50
Ap_4_A	P_i_ sensor	7.6	APase	ND	ND	57 ± 6
P_i_ sensor	9.0	APase	ND	ND	110 ± 6
MM_0920	8-Oxo-dATP	P_i_ sensor	7.6	PPase	(9.6 ± 0.8) × 10^−4^	12 ± 3	78 ± 12
8-Oxo-dGTP	P_i_ sensor	7.6	PPase	(1.9 ± 0.2) × 10^−3^	13 ± 3	149 ± 25

*Note. T* = 37 °C except for data from Ref. [14], where *T* = 30 °C. F–S assay, Fiske–SubbaRow assay; ND, not determined because *k*_cat_/*K*_m_ was obtained from linear regression fitting.

a Calculated from data in Table 2 of Ref. [14].

b Calculated from data in Fig. 2A of Ref. [26].

## Footnotes

1 *Abbreviations used:* mRNA, messenger RNA; P_i_, phosphate; APase, alkaline phosphatase; PBP, phosphate binding protein; MDCC, *N*-(2-(1-maleimidyl)ethyl)-7-(diethylamino)coumarin-3-carboxamide; P_i_ sensor, PBP labeled with MDCC; SDS–PAGE, sodium dodecyl sulfate–polyacrylamide gel electrophoresis; DMF, *N,N*-dimethylformamide; PPase, inorganic pyrophosphatase; CoA, coenzyme A.

## References

[1] Bessman M.J., Frick D.N., O’Handley S.F. (1996). The MutT proteins or “Nudix” hydrolases, a family of versatile, widely distributed, “housecleaning” enzymes. J. Biol. Chem..

[2] McLennan A. (2006). The Nudix hydrolase superfamily. Cell. Mol. Life Sci..

[3] Punta M., Coggill P.C., Eberhardt R.Y., Mistry J., Tate J., Boursnell C., Pang N., Forslund K., Ceric G., Clements J., Heger A., Holm L., Sonnhammer E.L.L., Eddy S.R., Bateman A., Finn R.D. (2011). The Pfam protein families database. Nucleic Acids Res..

[4] Mildvan A.S., Xia Z., Azurmendi H.F., Saraswat V., Legler P.M., Massiah M.A., Gabelli S.B., Bianchet M.A., Kang L.-W., Amzel L.M. (2005). Structures and mechanisms of Nudix hydrolases. Arch. Biochem. Biophys..

[5] Yang Q., Coseno M., Gilmartin G.M., Doublié S. (2011). Crystal structure of a human cleavage factor CFIm25/CFIm68/RNA complex provides an insight into poly(A) site recognition and RNA looping. Structure.

[6] Duong-Ly K.C., Gabelli S.B., Xu W., Dunn C.A., Schoeffield A.J., Bessman M.J., Amzel L.M. (2011). The Nudix hydrolase CDP-chase, a CDP-choline pyrophosphatase, is an asymmetric dimer with two distinct enzymatic activities. J. Bacteriol..

[7] Hahn F.M., Xuan J.W., Chambers A.F., Poulter C.D. (1996). Human isopentenyl diphosphate:dimethylallyl diphosphate isomerase: Overproduction, purification, and characterization. Arch. Biochem. Biophys..

[8] Huang N., De Ingeniis J., Galeazzi L., Mancini C., Korostelev Y.D., Rakhmaninova A.B., Gelfand M.S., Rodionov D.A., Raffaelli N., Zhang H. (2009). Structure and function of an ADP-ribose-dependent transcriptional regulator of NAD metabolism. Structure.

[9] Perraud A.-L., Fleig A., Dunn C.A., Bagley L.A., Launay P., Schmitz C., Stokes A.J., Zhu Q., Bessman M.J., Penner R., Kinet J.-P., Scharenberg A.M. (2001). ADP-ribose gating of the calcium-permeable LTRPC2 channel revealed by Nudix motif homology. Nature.

[10] Rodionov D.A., De Ingeniis J., Mancini C., Cimadamore F., Zhang H., Osterman A.L., Raffaelli N. (2008). Transcriptional regulation of NAD metabolism in bacteria: NrtR family of Nudix-related regulators. Nucleic Acids Res..

[11] Xia Z., Azurmendi H.F., Lairson L.L., Withers S.G., Gabelli S.B., Bianchet M.A., Amzel L.M., Mildvan A.S. (2005). Mutational, structural, and kinetic evidence for a dissociative mechanism in the GDP-mannose mannosyl hydrolase reaction. Biochemistry.

[12] Legler P.M., Lee H.C., Peisach J., Mildvan A.S. (2002). Kinetic and magnetic resonance studies of the role of metal ions in the mechanism of *Escherichia coli* GDP-mannose mannosyl hydrolase, an unusual Nudix enzyme. Biochemistry.

[13] Maki H., Sekiguchi M. (1992). MutT protein specifically hydrolyses a potent mutagenic substrate for DNA synthesis. Nature.

[14] Ito R., Hayakawa H., Sekiguchi M., Ishibashi T. (2005). Multiple enzyme activities of *Escherichia coli* MutT protein for sanitization of DNA and RNA precursor pools. Biochemistry.

[15] Ames B.N., Dubin D.T. (1960). The role of polyamines in the neutralization of bacteriophage deoxyribonucleic acid. J. Biol. Chem..

[16] Brune M., Hunter J.L., Corrie J.E., Webb M.R. (1994). Direct, real-time measurement of rapid inorganic phosphate release using a novel fluorescent probe and its application to actomyosin subfragment 1 ATPase. Biochemistry.

[17] Xu W., Shen J., Dunn C.A., Desai S., Bessman M.J. (2001). The Nudix hydrolases of *Deinococcus radiodurans*. Mol. Microbiol..

[18] Harris T.K., Wu G., Massiah M.A., Mildvan A.S. (2000). Mutational, kinetic, and NMR studies of the roles of conserved glutamate residues and of lysine-39 in the mechanism of the MutT pyrophosphohydrolase. Biochemistry.

[19] Legler P.M., Massiah M.A., Bessman M.J., Mildvan A.S. (2000). GDP-mannose mannosyl hydrolase catalyzes nucleophilic substitution at carbon, unlike all other Nudix hydrolases. Biochemistry.

[20] Sauder M.J., Rutter M.E., Bain K., Rooney I., Gheyi T., Atwell S., Thompson D.A., Emtage S., Burley S.K., Kobe B., Guss M., Huber T. (2008). High throughput protein production and crystallization at NYSGXRC. Structural Proteomics.

[21] Brune M., Hunter J.L., Howell S.A., Martin S.R., Hazlett T.L., Corrie J.E.T., Webb M.R. (1998). Mechanism of inorganic phosphate interaction with phosphate binding protein from *Escherichia coli*. Biochemistry.

[22] Bhatnagar S.K., Bessman M.J. (1988). Studies on the mutator gene, mutT of *Escherichia coli:* Molecular cloning of the gene, purification of the gene product, and identification of a novel nucleoside triphosphatase. J. Biol. Chem..

[23] Frick D.N., Townsend B.D., Bessman M.J. (1995). A novel GDP-mannose mannosyl hydrolase shares homology with the MutT family of enzymes. J. Biol. Chem..

[24] E. Gasteiger, C. Hoogland, A. Gattiker, S. Duvaud, M.R. Wilkins, R.D. Appel, A. Bairoch, Protein identification and analysis tools on the ExPASy server, in: J.M. Walker (Ed.), The Proteomics Protocols Handbook, Humana Press, Totowa, NJ, n.d., 2005, pp. 571–607.

[25] Webb M.R., Johnson K.A. (2003). A fluorescent sensor to assay inorganic phosphate. Kinetic Analysis of Macromolecules: A Practical Approach.

[26] Legler P.M., Massiah M.A., Mildvan A.S. (2002). Mutational, kinetic, and NMR studies of the mechanism of *E. coli* GDP-mannose mannosyl hydrolase, an unusual Nudix enzyme. Biochemistry.

[27] Saraswat V., Massiah M.A., Lopez G., Amzel L.M., Mildvan A.S. (2002). Interactions of the products, 8-oxo-dGMP, dGMP, and pyrophosphate with the MutT nucleoside triphosphate pyrophosphohydrolase. Biochemistry.

[28] Holm L., Rosenstrom P. (2010). Dali server: Conservation mapping in 3D. Nucleic Acids Res..

[29] Pegan S.D., Tian Y., Sershon V., Mesecar A.D. (2010). A universal, fully automated high throughput screening assay for pyrophosphate and phosphate release from enzymatic reactions. Comb. Chem. High Throughput Screen..

[30] Ofman R., Speijer D., Leen R., Wanders R.J.A. (2006). Proteomic analysis of mouse kidney peroxisomes: Identification of RP2p as a peroxisomal nudix hydrolase with acyl-CoA diphosphatase activity. Biochem. J..

[31] Cartwright J.L., Britton P., Minnick M.F., McLennan A.G. (1999). The IalA invasion gene of *Bartonella bacilliformis* encodes a (di)nucleoside polyphosphate hydrolase of the MutT motif family and has homologs in other invasive bacteria. Biochem. Biophys. Res. Commun..

[32] Lieb M. (1991). Spontaneous mutation at a 5-methylcytosine hotspot is prevented by very short patch (VSP) mismatch repair. Genetics.

[33] O’Handley S.F., Dunn C.A., Bessman M.J. (2001). Orf135 from *Escherichia coli* is a Nudix hydrolase specific for CTP, dCTP, and 5-methyl-dCTP. J. Biol. Chem..

